# DNA Packaging Specificity of Bacteriophage N15 with an Excursion into the Genetics of a Cohesive End Mismatch

**DOI:** 10.1371/journal.pone.0141934

**Published:** 2015-12-03

**Authors:** Michael Feiss, Jea Young Min, Sawsan Sultana, Priyal Patel, Jean Sippy

**Affiliations:** Department of Microbiology, Carver College of Medicine, University of Iowa, Iowa City, Iowa, 52242, United States of America; Centro Nacional de Biotecnologia - CSIC / CIF Q2818002D, SPAIN

## Abstract

During DNA replication by the λ-like bacteriophages, immature concatemeric DNA is produced by rolling circle replication. The concatemers are processed into mature chromosomes with cohesive ends, and packaged into prohead shells, during virion assembly. Cohesive ends are generated by the viral enzyme terminase, which introduces staggered nicks at *cos*, an approx. 200 bp-long sequence containing subsites *cosQ*, *cosN* and *cosB*. Interactions of *cos* subsites of immature concatemeric DNA with terminase orchestrate DNA processing and packaging. To initiate DNA packaging, terminase interacts with *cosB* and nicks *cosN*. The cohesive ends of N15 DNA differ from those of λ at 2/12 positions. Genetic experiments show that phages with chromosomes containing mismatched cohesive ends are functional. In at least some infections, the cohesive end mismatch persists through cyclization and replication, so that progeny phages of both allelic types are produced in the infected cell. N15 possesses an asymmetric packaging specificity: N15 DNA is not packaged by phages λ or 21, but surprisingly, N15-specific terminase packages λ DNA. Implications for genetic interactions among λ-like bacteriophages are discussed.

## Introduction

Large DNA viruses, such as tailed bacteriophages and herpes viruses, use an ATP-powered motor to translocate viral DNA into the preformed empty shell, called the prohead or procapsid [1–3]. Recent structural and bioinformatic studies demonstrate that the DNA packaging machinery of these viruses is descended from that of an ancient common ancestor [4]. For example, the prohead shell is an icosahedral lattice principally constructed of many copies of the major capsid protein whose fold is conserved [5]. Similarly, one of the prohead’s 5-fold vertexes, the unique portal vertex, contains the radially disposed dodecameric portal protein. The portal protein contains a channel through which DNA enters and exits the shell interior [6–9]. Terminase, also conserved in the herpes viruses and tailed bacteriophages, is usually a hetero-oligomer of small (TerS) and large (TerL) subunits [2,10–12]. TerS carries out viral DNA recognition. TerL is a motor protein whose N-terminal ATPase domain powers translocation of the DNA into the prohead. TerL also contains the C-terminal endonuclease domain that cuts concatemeric DNA into unit-length virion chromosomes.

TerS molecules contain three domains, as follows. An α-helical central domain oligomerizes TerS into cylindrical, 8- to 12-mer oligomers, depending on the virus. At the C-terminus is a β-barrel structure. The C-terminus contains a functional TerL binding domain, as shown for λ and 21 [13,14], P22 [15,16], and T4 [17,18]. In the case of P22’s TerL, short α-helical extensions at the extreme C-terminus may tether TerS to TerL [19]. At the TerS N-terminus is a DNA binding motif. In the cases of λ [20], Sf6 [21], T4 [18,22], and SF6 [23,24], the DNA binding motif is likely a classic helix-turn-helix. For SF6 TerS, the DNA binding domains are tethered to the central cylinder by linkers hence are highly mobile. It is proposed in a number of these cases that the DNA is wrapped around the TerS oligomer into a nucleosome-like structure by interactions with multiple TerS DNA binding domains [15,17,25,26]. Phage λ TerS is unusual in that in addition to oligomerizing, the DNA binding domain forms a tight dimer [20], as discussed further below.

Among the tailed dsDNA bacteriophages, *pac* phages use a headful mechanism to package virion DNA from concatemers, i.e., immature end-to-end multimers of virus DNA produced by rolling circle replication. An initial cut is made near a *pac* site, the TerS recognition site, and packaging is terminated by a non-specific cut when the prohead is full. The first termination cut is also the start of packaging of the next chromosome along the concatemer, as packaging is processive. The resulting viral chromosomes have a terminal redundancy, and individual chromosomes are circular permutations of the unique viral sequence. In contrast, virion DNAs of *cos* phages have a unique DNA sequence with short complementary cohesive ends. *cos* phages produce virion DNAs through TerL’s introduction of precisely staggered nicks in concatemeric DNA. The interaction of TerS with *pac* or *cos* recognition sites varies from phage to phage. *pac* phage P22’s TerS recognition site is a simple, 22 bp, asymmetric sequence located in the TerS gene [27]. In contrast, for *pac* phage SPP1, there are multiple TerS binding sites flanking the site where the initial cut is made [25]. For T4, the headful mechanism is used, but initiation is complex and not well understood [28].

For *cos* phage λ and its close relatives, virion chromosomes have cohesive ends, i.e., complementary, 5’-ended, 12 base-long, single-strand extensions that anneal, circularizing the DNA, upon entry into a host cell. The DNA segment containing the DNA packaging signals and the annealed cohesive ends is called *cos*. At late times during the lytic cycle, recombination and rolling circle replication produce concatemeric DNA [29]. During packaging, concatemeric DNA is recognized and processed by terminase into monomeric virion chromosomes. TerS^λ^ and TerL^λ^ are gene products of the *Nu1* and *A* genes, respectively. TerS^λ^’s winged helix-turn-helix (wHTH) domain, which specifically binds *cos*, forms a tight dimer [20]. The simplest form of λ terminase is the protomer, a TerS_2_:TerL_1_ heterotrimer. Protomers further assemble into [TerS_2_:TerL_1_]_4_ tetramers [30,31]. TerS^λ^ recognizes λDNA by specific binding to *cosB*, a *cos* subsite adjacent to *cosN*, the nicking site at which TerL^λ^ introduces staggered nicks to generate the cohesive ends of mature virion chromosomes. Assembly of TerS^λ^ on *cosB* ^*λ*^ positions TerL^λ^’s endonuclease to introduce the staggered nicks at *cosN* [32–34]. When *cosB* is deleted or re-positioned, *cosN* nicking is inefficient and inaccurate, indicating that anchoring by TerS is critical for nick introduction by TerL [33–36].

Packaging is initiated when terminase binds and nicks a *cos* on a concatemer. Following *cosN* nicking and cohesive end separation, terminase forms a tight complex, Complex I, on the *cosB*-containing chromosomal end [37,38]. Complex I then docks on the portal protein, gpB, of the prohead [39,40]. Following docking of Complex I on the portal, TerL^λ^’s ATPase is activated and ATP hydrolysis-powered translocation of the DNA into the shell ensues [41,42]. Terminase remains docked on the portal during translocation. When the next *cos* along the concatemer is encountered, terminase (1) nicks *cosN*, (2) dissociates from the portal, and (3) remains bound to the *cosB*-containing end of the next chromosome along the concatemer [43]. By remaining bound to the next chromosome along the concatemer as a Complex I, terminase processively packages downstream chromosomes. A third sub-site, *cosQ*, is essential for recognition and nicking of the downstream *cos* during termination of packaging [44,45]. In sum, *cosN* and *cosB* are required to initiate λDNA packaging, *cosQ* and *cosN* are required for termination, and all three subsites are required for processivity [46].

**Fig 1:**
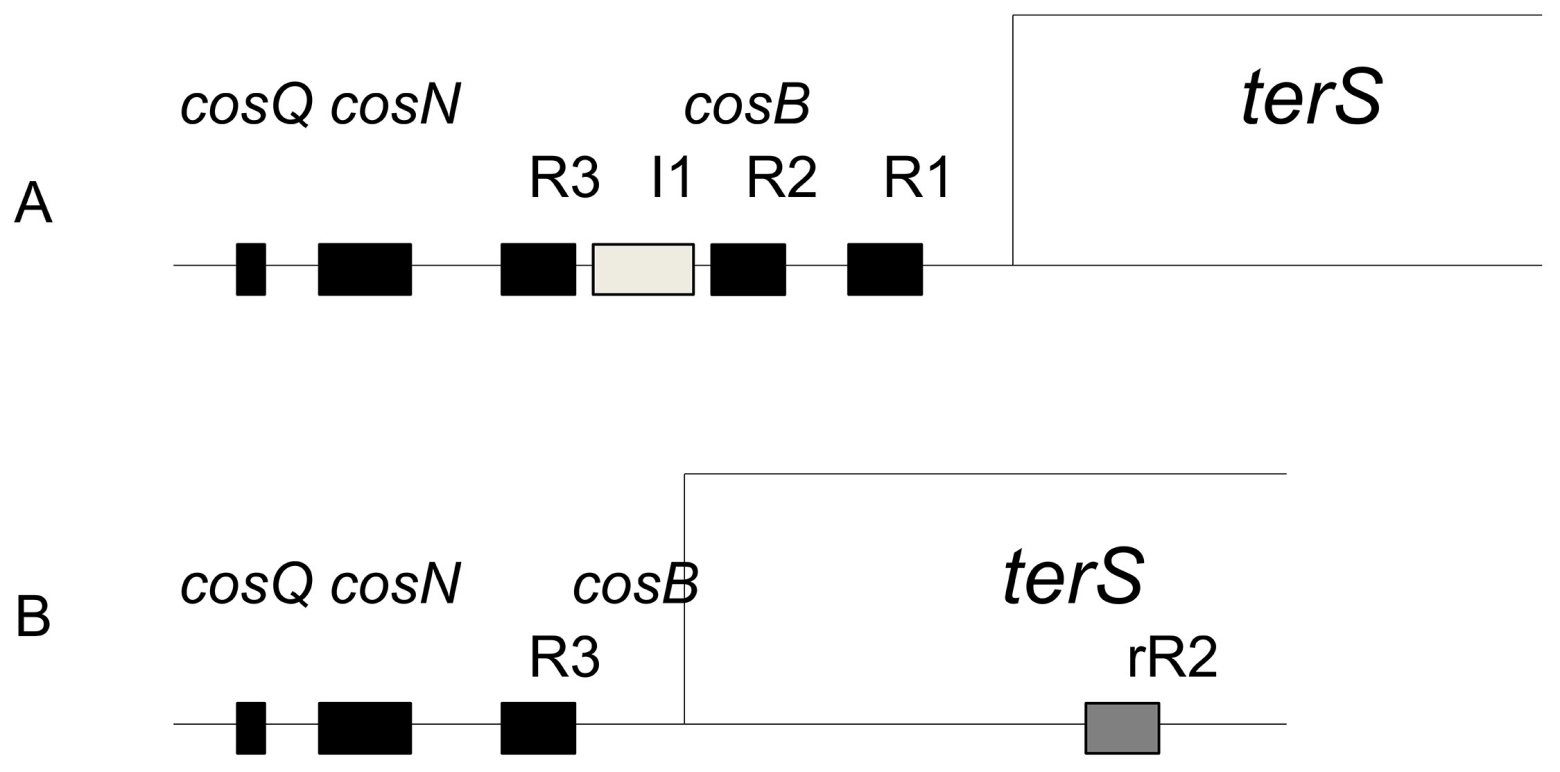
Comparison of the *cos*es and terminase small subunit genes of λ, 21 and N15. A. Complex *cos* structure of phages λ, 21, Monarch. R3, R2 and R1 sequences are TerS binding sites, and I1 is an IHF binding site. B. Simple *cos* of N15 and relatives [58]. rR2 is a proposed accessory TerS binding site in the *cos* of N15 and relatives.

The 120 bp-long *cosB* ^λ^ is complex, containing three TerS binding sites, R3, R2, and R1 (Fig 1) [38]. Between R3 and R2 is a binding site [47], I1, for IHF, the *E*. *coli* site-specific DNA bending protein [48–51]. IHF bends DNA into an approx. 180° hairpin [52]. At *cos*, the IHF-induced bend at I1 positions R3 and R2 such that the wHTH motifs of dimeric TerS can be docked into the major grooves [20]. Complex I likely includes this nucleoprotein structure [53].

In this paper, we refer to the λ-like phages as those with λ-like cohesive ends, i.e., 12 base-long, 5’ extensions with significant identity to λ's cohesive ends. This group includes λ, 434, ϕ80, 21, N15, Monarch, and gifsy-1 [54]. In the *cosN*s of this group, bp differences in the right half of the cohesive end sequences, i.e., bp 7–12, are common. For example N15’s cohesive ends differ from λ’s at bp 9 and 12, and gifsy-1’s differ at positions 8, 9, and 11. In contrast, the 7 bp *cosQ* site is highly conserved. The *cosB*s of the λ-like phages differ both in structural complexity and in packaging specificity, as follows.

With respect to *cosB* structure, DNA sequence analysis suggest that many λ-like phages have a complex *cosB* similar to *cosB* ^*λ*^, with three R sequences with R2 oriented opposite to R3 and R1, and with a likely IHF binding site between R3 and R2. Recently, N15 was shown to have a different, simpler *cosB* consisting of a single critical TerS binding site located approximately at the position of the R3 in complex *cosB*s. A second, inverted sequence that closely matches R3^N15^, called rR2, is located within the TerS gene, about 200 bp distal to R3^N15^, and though not critical for DNA packaging, is proposed to play a non-essential, accessory role in DNA packaging. The R1 sequence of *cosB* ^*λ*^ plays such a role [55]. Examination of prophages in Genebank identified an additional five prophages that have the R3 and rR2 sequences found in N15’s *cosB* [56]. In sum, two strikingly different *cosB* structures, complex and simple, are found in the λ-like phages.

**Fig 2:**
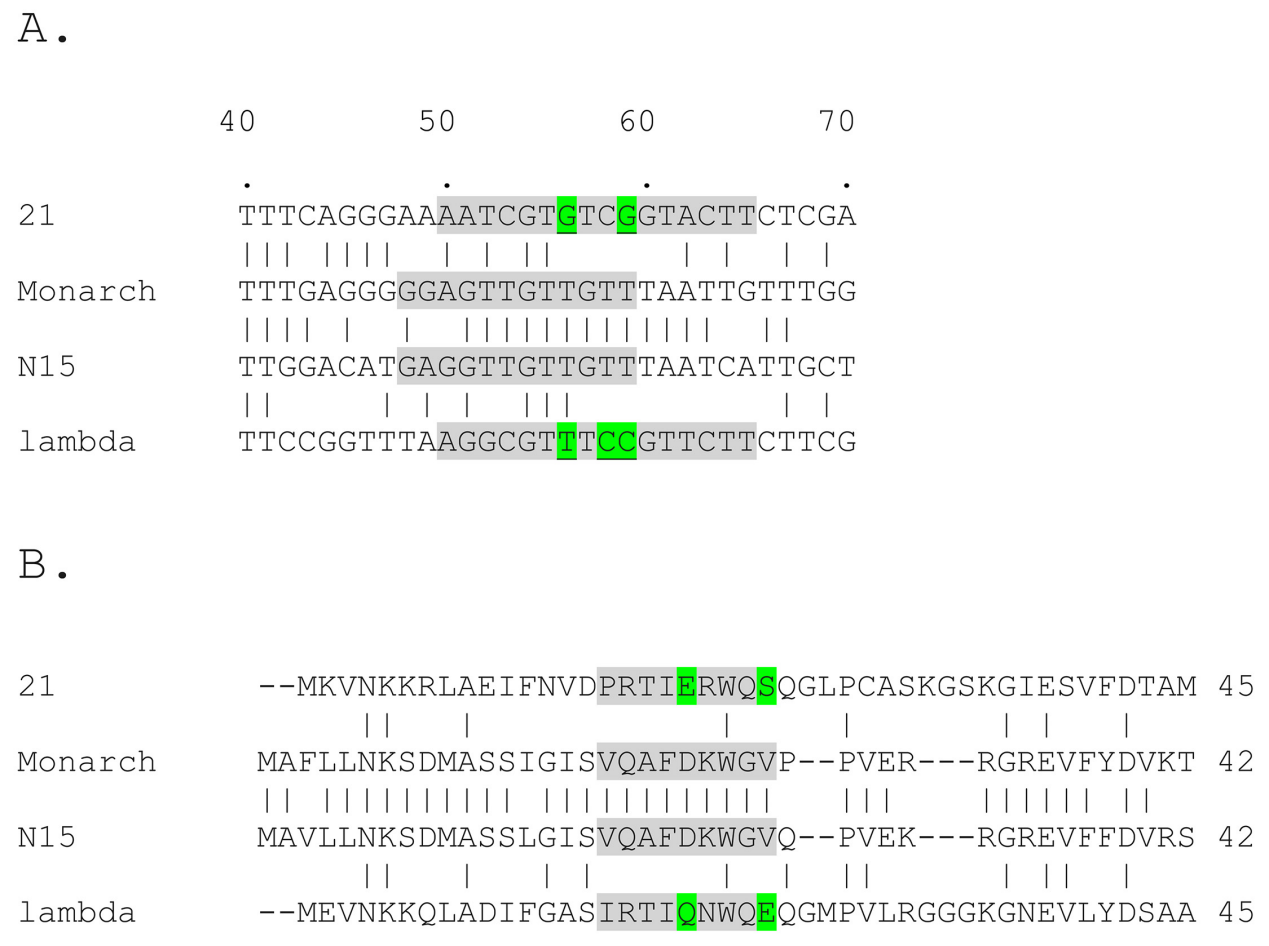
Alignments of the *cosB* and TerS DNA packaging recognition elements of λ-like bacteriophages. A. Alignment of R3 sequences of 21, Monarch, N15, and λ. Numbering is rightwards from the first base of the left cohesive end of each phage. Putative R3 sequences are highlighted in grey. The boundaries and locations of the R3 elements are only approximate and are mostly based on sequence conservation among the R3, R2 and R1 elements. Residues 56 and 59 in R3^λ^ and R3^21^ are important for recognition by TerS and are highlighted in green [56]. The CG→TA change at R3^λ^ bp 58 (highlighted in green) renders λ IHF-dependent for plaque formation [49,70][62]. B. Alignments of the left ends of TerS subunits. Proposed recognition helixes are highlighted in grey and residues 20 and 24 of TerS^21^ and TerS^λ^, known to be involved in packaging recognition and discrimination, are highlighted in green.

In addition to structural differences, packaging specificity differences are found in the λ-like phages, as exemplified by the well-characterized specificities of λ and 21. λ and 21 have identical *cosQ* and *cosN* sequences, yet each package self-DNA >10^3^-fold more efficiently than that of the other phage. Examination of the recognition helix and *cosB* R site sequences shows significant differences that account for the specificity difference (Fig 2) [20,57]. Genetic analysis identified recognition helix:R site contacts that differ between λ and 21, plus clashes involved in discrimination by TerS^λ^ and TerS^21^ against the heterologous DNA [56]. Looking at TerS^N15^ and R3^N15^ suggests that N15, and its relatives with simple *cosB*s might have a novel packaging specificity distinct from the λ and 21 specificities (Fig 2)[58]. Interestingly, a second group of N15-like prophages, exemplified by Monarch, have complex, tripartite *cosB*s. TerS^Monarch^ and TerS^N15^ have identical recognition α-helixes and strong R site identity, suggesting that both subgroups share the same packaging specificity. Here we present a study of N15’s packaging specificity. Because of the strategy used, a preliminary analysis of the genetics of phage chromosomes with mismatched cohesive ends, λ versus N15, was carried out.

## Results

### Genetics of a cohesive end mismatch: Phages with mismatched cohesive ends are viable; the mismatches can persist to post-DNA replication

#### Strategy for studying DNA packaging specificity

**Fig 3:**
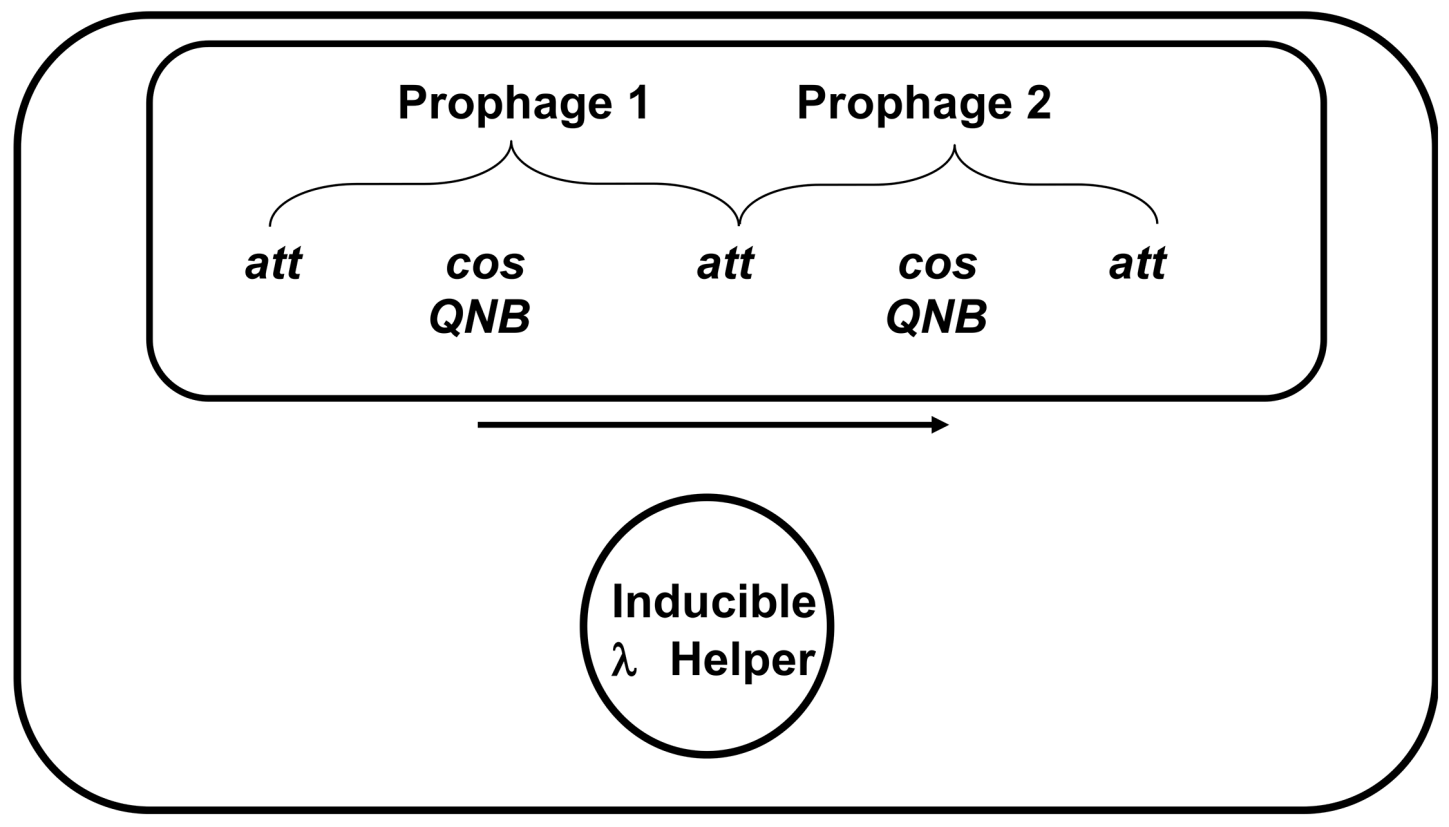
Helper packaging strategy. The large and small rectangles represent the envelope and chromosome of an *E*. *coli* cell with three prophages. Prophages1 and 2 are inserted in tandem in the bacterial chromosome, and the circle represents a third, heat-inducible plasmid prophage, the helper prophage. Induction of the helper prophage to lytic growth provides packaging components for both helper DNA and prophage DNA, provided the helper’s terminase can initiate packaging at the *cos* of upstream prophage 1 and terminate packaging at the *cos* of downstream prophage 2. The DNA packaged from the tandem prophages is represented by the horizontal arrow. The tandem prophages are not heat-inducible and remain repressed during the experiment.

To ask about the effects of *cosN* and *cosB* differences on DNA packaging, we used a helper phage/passive prophage approach (Fig 3). In these experiments, the DNA packaging substrate, a passive prophage, is provided to the DNA packaging machinery of a lytically growing “helper” phage, as follows. Two prophages integrated in tandem at *attB* contain between them a chromosome that mimics a chromosome in a concatemer. Terminase from a helper phage can initiate packaging at the *cos* of the upstream prophage, translocate DNA and terminate packaging at the *cos* of the downstream prophage. In our experiments, the helper phage was a heat-inducible (*cI857*) derivative of a λ strain that forms a plasmid prophage. Helpers with λ, 21 or N15 DNA packaging specificity were used. The 21-and N15-specific helpers were viable hybrid derivatives of λ in which a left chromosome end, the DNA segment including *cosB* ^*λ*^ and much of the TerS^λ^-encoding *Nu1* gene, was replaced with the functionally analogous segment from phages 21 or N15, respectively. The 21- and N15-specific helpers are called λ21^hy51^ [59] and λN15^hy4^[58], respectively. These helper phages are viable hybrid phages in which the left λ DNA end is replaced with the corresponding left end of the 21 and N15 chromosome, respectively. The left end segments, each about 600 bp in length, include 21’s or N15’s *cosB* and the 5’ end of the TerS-encoding gene which contains the helix-turn-helix DNA binding specificity motif. The corresponding chimeric TerS proteins are called TerS^hy51^ and TerS^hy4^, respectively. When a helper prophage is heat-induced to carry out lytic growth, the tandem prophages remain repressed because the prophages have thermo-stable *imm^21^*and *imm^434^* immunity repressors that differ in specificity from the *imm^λ^* helper. The maximum number of packageable passive prophages equals the number of bacterial chromosomes in the cell, so efficient packaging of a prophage gives a yield of 1–4 virions/infected cell; the helper yield was also low, at ~10–20/induced lysogen.

#### Genetics of mismatched cohesive ends

**Fig 4:**
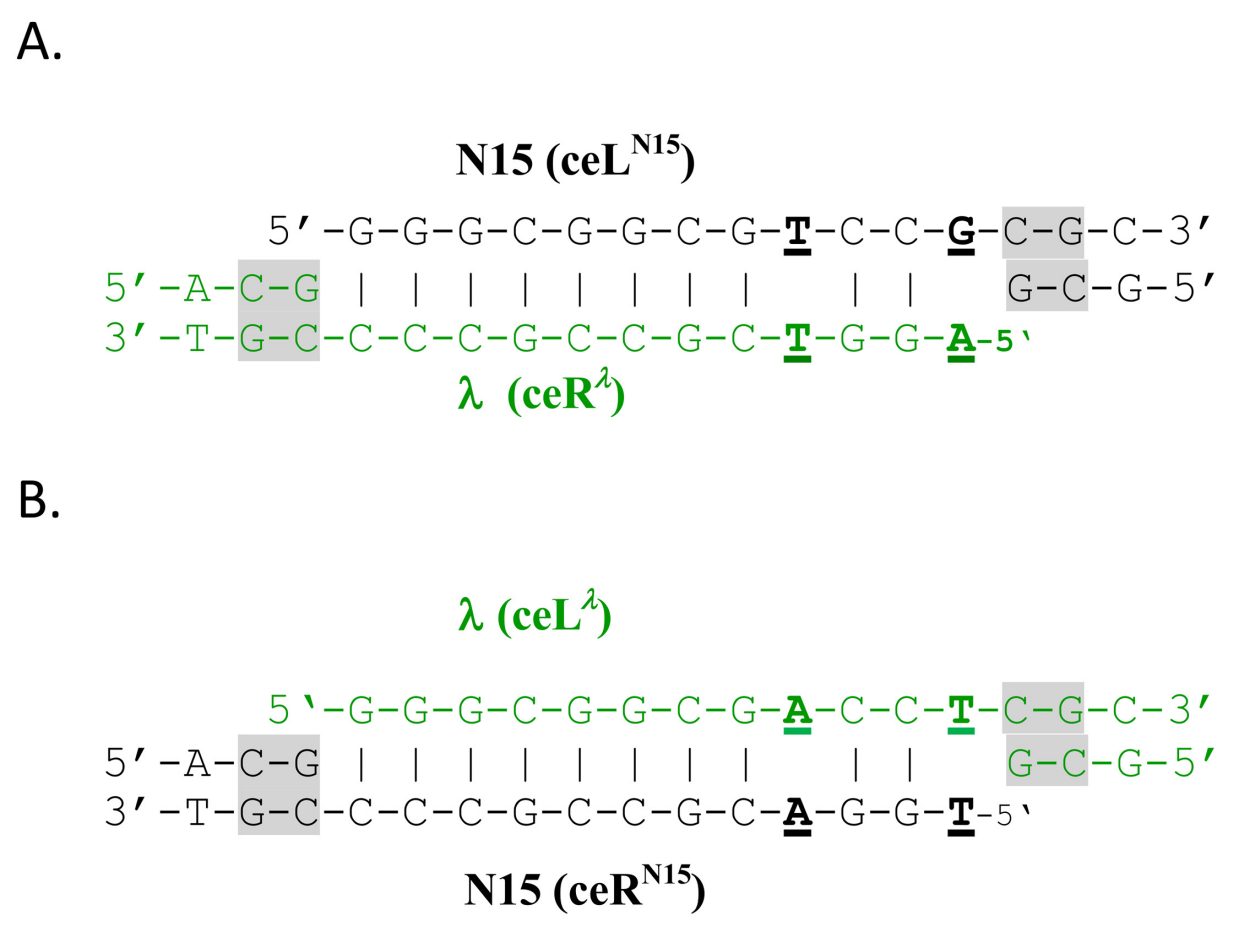
Annealed cohesive ends of phage chromosomes with cohesive end mismatches. A. The mismatch of a phage DNA with a ceL^N15^/ceR^λ^ mismatch. B. The mismatch of a phage DNA with a ceL^λ^/ceR^N15^ mismatch. Mismatched bp are underlined and in bold. Highly conserved bp flanking the nick sites are highlighted in grey.

We did preliminary helper packaging experiments asking if the helper packaging approach was appropriate for N15 specificity studies. First, using *in vivo* phage crossing techniques, we made λ *att* ^*+*^ N15^hy4^, phage ϕ1182 (Table 1), for dilysogen construction. ϕ1182 was used to lysogenize MF532, which contains a partial λ *imm* ^*434*^ *ind* ^*-*^prophage which includes the prophage segment from *attL* through *cos* ^*λ*^. MF532 (ϕ1182) contains the passive prophage structure: *imm^21^ cosQ^N15^N^N15^B^N15^* → *imm^434^ ind* ^*-*^ *cosQ^λ^N^λ^B^λ^*, where the arrow indicates the direction of DNA packaging. Packaging of the passive prophage by the λ N15^hy4^ helper was expected to package a passive chromosome with mismatched cohesive ends. That is, initiation at the *cos^N15^* of the upstream prophage and termination at the *cos^λ^* of the downstream prophage generates a helper-packaged chromosome with an N15 left cohesive end (designated as ceL^N15^) and a λ right cohesive end (ceR^λ^; Fig 4). It was uncertain whether phages carrying the mismatched passive chromosome would be viable. Would the chromosome with a 2-bp cohesive end mismatch be able to circularize? Would the λ N15^hy4^ terminase, after initiating packaging at the *cos* ^*N15*^ of the upstream prophage, be able to terminate packaging at the *cos* ^*λ*^ of the downstream prophage? Although it was unknown whether N15-specific terminase would terminate packaging at *cos* ^*λ*^, previous work showed that λ and 21 terminases could terminate packaging at either *cos* ^*λ*^ or *cos* ^*21*^. In fact, previous work indicated that termination appears to not depend on the presence of *cosB* [46]. Another termination issue was whether the helper’s N15-specific terminase would show preference for the downstream *cosN* sequence. Previous work demonstrated that TerL^λ^ efficiently nicks a *cosN* with bp changes in the right half of the cohesive end sequence [60], indicating *cosN* specificity was also not a likely issue.

**Table 1:** Bacteria and Phages.

Strain/Plasmid	Genotype/Comments	Source/Reference
A. Bacteria		
MF713	λ *b511* Δ_SA509_[*gal-attL-S*], *recA1*	[71]
MF532	λ *imm^434^ ind* ^-^ Δ_SA441_[*Nu1-attR-bio-uvrB-chlA*] *recA1*	[71]
MF611	W3101 *recA1*	[61]
MF3510	MF611 *galK103*	Feiss collection
C600	*thi leu thr supE* / host for plaque assays	Our collection
C600 (λ *Bam1*)	*thi leu thr supE* / Kn transduction recipient	Feiss collection
MF1427	C1a *galK*	
B. Phages		
λ-P1:5R Kn^R^ *cI857 nin5* = ϕ1080	Helper phage with λ packaging specificity	[55]
λ-P1:5R Kn^R^ 21^hy51^ *cI857 nin5* = ϕ1081	Helper phage with 21 packaging specificity	[64]
λ-P1:5R Kn^R^ N15^hy4^ *cI857 nin5* = ϕ1187	Helper phage with N15 packaging specificity	[58]
λ-P1:5R Kn^R^ *cI857 nin5 Nu1ms1* = ϕ1082	Helper phage	[55,62]
λ-P1:5R Kn^R^ *cI857 nin5 cos2*	*cosN* deletion phage	[55]
λ *imm* ^*21*^ = ϕ72	*cos^λ^* / dilysogen constructions	Feiss collection
λ N15^hy4^ *cosQ* ^*N15*^ *cosN* ^*N15*^ *cosB* ^*N15*^ *imm* ^*21*^ *red3 =* ϕ1182	*cos ^N15^*/ dilysogen constructions	This work
λ N15^hy4^ *cosQ^λ^N^λ^B^N15^ imm* ^*434*^ *ind^-^* = ϕ1197	Dilysogen constructions	This work
λ N15^hy4^ *cosQ* ^*λ*^ *N* ^*N15*^ *B* ^*N15*^ *imm* ^*434*^ *ind^-^* = ϕ1199	Dilysogen constructions	This work
λ *cosQ^λ^N^N15^B^λ^imm* ^*434*^ *ind* ^*-*^ *b511* = ϕ1201	Dilysogen constructions	This work
λ *cosQ^λ^N^λ^B^λ^imm* ^*434*^ *ind* ^*-*^ *b511* = ϕ1203	Dilysogen constructions	This work
λ 21^hy33^ *gal^+^ att^+^ imm^21^* ϕ1221	*cos^21^*/ dilysogen constructions	This work
λ *gal^+^ att^+^ imm^21^* = ϕ1225	*cos^λ^*/ dilysogen constructions	This work
λ N15^hy4^ *cosN* ^*λ*^ *imm* ^*21*^ *gal^+^ att^+^* = ϕ1227	Passive prophage with N15 packaging specificity	This work
λ *cI*857 Δ(*stf-tfa*)::*cat* = ϕ1208	*cat* gene provides resistance to chloramphenicol	[65]
λ *imm^434^ ind^-^* Δ(*stf-tfa*)::*cat* = ϕ1220	Passive *cos* ^*λ*^ prophage	This work

Packaging of MF532 (ϕ1182)’s passive prophage by the λN15^hy4^ helper produced a modest yield (0.1 phages/induced cell) bearing passive prophages with mismatched cohesive ends. This yield is about 10% of the yield one might expect. The low yield of this ceL^N15^ → ceR^λ^ chromosome might be due to inefficiency at any of the stages of DNA packaging, i.e., initiation, termination, or reduced viability of the virions carrying chromosomes with mismatched cohesive ends, or the infirm *recA* ^*-*^ *uvrB* ^*-*^ host cells. Plaque-formation by ceL^N15^/ceR^λ^ mismatch phages indicates that the cohesive end mismatch is not lethal, i.e., the mismatch does not block cyclization of the injected DNA. Finding that phages with mismatched cohesive ends are viable indicated that none of the confounding possibilities prevented packaging of mismatched chromosomes into virions that were competent to carry out successful infections. Below we report experiments directly testing both *cosN* specificity and termination efficiency of λ- and N15-specific terminases at heterologous *cos*es.

The cyclized DNA of the mismatch phages from MF532 (ϕ1182) contains a *cosN* with mismatches at positions 9 and 12 (Fig 4A). The mismatched bp might or might not be subject to repair prior to DNA replication. If the mismatches are repaired, then all the progeny of an infection by a mismatch phage will either be *cosN* ^*λ*^ or *cosN* ^*N15*^, depending on the direction of the repair process. If the *cosN* mismatch is not repaired, then a mixed burst of both *cosN* ^*λ*^ and *cosN* ^*N15*^ progeny phages are expected from an infected cell. To ask if the mismatched bp were or were not repaired, four well-isolated plaques produced by mismatch phages were suspended in buffer, replated, and individual plaques were subjected to DNA sequence analysis to determine the cohesive end sequence. We found that in the progeny of two of the four infections by mismatch phages, both *cosN* ^*λ*^ and *cosN* ^*N15*^ phages were present (Table 2). We conclude that in at least some infections, the mismatch is not repaired and DNA replication produces a progeny phage burst in which some are *cosN^λ^*and some are *cosN^N15^*. In practical terms, the MF532 (ϕ1182) helper packaging produced phages that were *imm* ^*434*^ *ind* ^*-*^ *cosQ* ^*λ*^ *cosB* ^*N15*^ and either *cosN* ^*λ*^ (ϕ1197) or *cosN* ^*N15*^ (ϕ1199).

**Table 2:** Both cohesive end alleles are found in the progeny of phages with mismatched cohesive ends.

	Parent Phage’s Cohesive End Mismatch			
	ceL^N15^– ceR^λ^		ceL^λ^ –ceR^N15^	
Plaque of phage with mismatched cohesive ends	Cohesive end alleles of progeny phages			
	λ	N15	λ	N15
1	10	0	10	0
2	10	0	9	0
3	6	4	11	0
4	9	1	11	0
5	—	—	1	10
Heteroallelic bursts total bursts	2/4		1/5	

The ceL^N15^-ceR^λ^ phages resulted from packaging of the *cosQ^N15^ N ^N15^ B ^N15^*→*cosQ^λ^N^λ^B^λ^* passive prophage of MF532 (ϕ1182) by the N15-specific helper, ϕ1187. The ceL^λ^-ceR^N15^ phages resulted from packaging of the *cosQ^λ^N^λ^B^λ^*→*cosQ^λ^N ^N15^B ^N15^* passive prophage of MF713 (ϕ1199) by the λ-specific helper, ϕ1080. Progeny of phages with mismatched cohesive ends were sampled from well-separated plaques on lawns of MF1427.

A second dilysogen, MF713 (ϕ1199), was constructed. MF711 contains a partial λ *b511* prophage containing the prophage segment extending rightward from *cos* ^*λ*^. MF713 (ϕ1199) has the prophage structure: *cosQ^λ^N^λ^B^λ^* →*cosQ* ^*λ*^ *N ^N15^B ^N15^*. Packaging MF713 (ϕ1199)’s passive prophage by the λ helper is expected to produce phages bearing chromosomes with the reciprocal mismatch, i.e., a ceL^λ^/ceR^N15^ mismatch (Fig 4B). Using the λ-specific helper, the yield was 0.67 phages bearing passive prophage chromosomes/induced cell, indicating that the reciprocal cohesive end mismatch is also not lethal. To ask about persistence of the mismatch, the progeny of five of these ceL^λ^/ceR^N15^ mismatch phages were examined. Four isolates produced only progeny with *cosN* ^*λ*^, and a fifth isolate produced a burst containing both *cosN^λ^*and *cosN^N15^* phages (Table 2). As was the case for ceL^N15^/ceR^λ^ mismatch phages, some ceL^λ^/ceR^N15^ mismatch phages give rise to both *cosN* ^*λ*^ phages and *cosN* ^*N15*^ phages. In practical terms, the MF713 (ϕ1199) helper packaging experiment produced a λ *imm434 ind* ^*-*^ *b511* phage pair with *cosQ* ^*λ*^ *cosB* ^*λ*^ and with either *cosN* ^*λ*^ (ϕ1203) or *cosN* ^*N15*^(ϕ1201) that were useful for dilysogen constructions for experiments presented below.

Although the data are limited, there is a preponderance of *cosN* ^*λ*^ progeny; the reason for this asymmetry is unknown. In both orientations, both cohesive end alleles are found in progeny phages, indicating that at least in some infections, mismatches are not resolved when the cohesive ends anneal and are ligated. Rather the mismatch persists until the mismatch is resolved by DNA replication. In sum, the cohesive end mismatch does not block cyclization, and in at least some cases, the mismatch persists until after the mismatch-containing parental chromosome has replicated. The limited data also are consistent with the scenario that in some infections by a mismatch chromosome, repair may resolve the mismatch prior to replication.

#### Quantitating effects of a cohesive end mismatch on infectious phage production

To ask about the quantitative effects of a cohesive end mismatch on infectious phage production, MF4942, with the prophage structure *imm* ^*21*^ *cosQ^λ^N^λ^B^λ^*→ λ *imm^434^ cosQ^λ^ N* ^*N15*^ *B*, and MF4943, with the prophage structure λ *imm^21^ cosQ^λ^N^λ^B^λ^*→ λ *imm^434^ cosQ^λ^N^λ^B^λ^*, were constructed and used in helper packaging experiments (see S1 Table for details). With the λ-specific helper, packaging the ceL^N15^/ceR^λ^ mismatched chromosome, the yield was about 25% that of a chromosome with matched ceL^λ^/ceR^λ^cohesive ends (Table 3, compare lines 1 and 3), indicating that the mismatch reduced the level of viable virion production by four-fold. Remarkably, the λ-N15^hy4^ helper phage was able to package λ DNA rather efficiently, at about 50% the efficiency of the λ-specific helper (Table 3, compare lines 1 and 2 and lines 3 and 4). As with the λ-specific helper, the yield of viable ceL^N15^/ceR^λ^ mismatched chromosome-carrying phages was about 25% that of phages with matched cohesive ends (Table 3, compare lines 2 and 4).

**Table 3:** Effect of a cohesive end mismatch on viable phages production.

Line	Helper Specificity	Passive Prophage *cos* Structure^a^	Prophage Yield [sem]^b^
1	λ	*Q^λ^N^λ^B^λ^* → *Q^λ^N^λ^B^λ^*	2.41 [0.61]
2	N15	*Q^λ^N^λ^B^λ^* → *Q ^λ^N^λ^B^λ^*	1.24 [0.09]
3	λ	*Q^λ^N^λ^B^λ^* → *Q ^λ^N* ^*N15*^ *B^λ^*	0.61 [0.10]
4	N15	*Q^λ^N^λ^B^λ^* → *Q ^λ^N^N15^B^λ^*	0.27 [0.038]

^a^ The host bacteria were MF4943 for lines 1 and 2, and MF4942 for lines 3 and 4.

^b^ sem is the standard error of the mean, calculated from 3 to 5 independent observations.

To summarize, the λ/N15 cohesive end mismatch reduced the recovery of viable packaged chromosomes roughly fourfold, and the N15-specific helper packaged λ-specific DNA at about 50% efficiency.

### N15’s Packaging Specificity

#### *cosN* specificity

One explanation for the reduction in yield for mismatched phages is that packaging is inefficient due to reduced efficiency of *cos* cleavage. For example, since both the λ and N15^hy4^ terminases have TerL^λ^, both terminases might cleave *cosN^λ^* more efficiently than *cosN^N15^*. Alternatively, the TerS^N15hy4^:*cosB* ^*λ*^ interaction might affect TerL^λ^ positioning and the efficiency of *cosN* cleavage. In either case, λ terminase might act more efficiently in phages with *cosB^λ^* than N15^hy4^ terminase. To investigate this point, the yields of phage pairs expressing λ or N15^hy4^ terminase, with *cosB^λ^* and either *cosN^λ^* or *cosN^N15^*, were determined. The yields were found not to depend on *cosN*: for both λ and λ N15^hy4^ phages, the yield did not significantly differ between *cosN^λ^* and *cosN^N15^* (Table 4). These results do not support the idea that λ- and N15-specific terminases are *cosN*-specific. An alternative explanation for the yield reduction caused by a cohesive end mismatch is that DNA packaging is fully efficient, but the infectivity of the virions with mismatched cohesive ends is reduced. The reduction in yield might be due to less efficient cyclization or a lethal event caused by attempted mismatch repair. Finding a lack of *cosN* specificity is not surprising, as follows. First, the λ and N15 TerLs are quite similar, with 64.9% identity and 78.5% similarity. Second, the base pair differences in the cohesive end sequences are located in the right half of the cohesive end sequence, where sequence changes do not have measurable effects on *cos* cleavage [60].

**Table 4:** The terminases of λ and λ N15^hy4^ do not show *cosN* specificity.

Line	Phage	Yield^a^ pfu/infected cell [sem]^b^
1	λ–N15^hy4^ *cosQ^λ^N^N15^B^N15^* [ϕ1199]	103 [13]
2	λ–N15^hy4^ *cosQ^λ^N^λ^B^N15^* [ϕ1197]	111 [12.5]
3	λ *cosQ^λ^N^λ^B^λ^ b511* [ϕ1203]	120 [8]
4	λ *cosQ^λ^N^N15^B^λ^ b511* [ϕ1201]	132 [17]

^a^ Host and plating bacterium was MF1427.

^b^ [sem] is the standard error of the mean for 3 or more independent observations.

#### Termination specificity

Previous work showed that λ- and 21-specific terminases efficiently terminate DNA packaging at the downstream *cos* regardless of *cosB* specificity [61]. Later work showed that *cosQ*, *cosN* and I2, the latter a segment between *cosN* and *cosB*, were required for efficient packaging termination [46]. To ask if λ and N15^hy4^ terminases were also *cosB*-non-specific for packaging termination, dilysogens were constructed in which the initial *cos* was *cosB* ^*N15*^ and the downstream *cos* was *cosB* ^*N15*^ (MF4944) or *cosB* ^*λ*^ (MF4945). In a second pair of dilysogens, the initial *cos* was *cosB^λ^*, and the downstream *cos* was either *cosB^λ^* (MF4946) or *cosB^N15^* (MF4947). These dilysogens were then further lysogenized with the λ- and N15-specific helpers and used in helper packaging experiments. Neither helper displayed any *cosB* specificity for termination at the downstream *cos* (Table 5). These results extend the earlier observations that packaging termination is not dependent on *cosB* specificity [46]. These results are not unexpected given the strong sequence conservation for *cosQ*, *cosN* and I2 in λ, 21 and N15.

**Table 5:** Packaging termination does not depend on *cosB* specificity^a^.

Line	Prophage Structure	Helper Phage Specificity	HelperYield (ϕ/cell)[sem]	Prophage Yield (ϕ/cell) [sem]^b^
1	*cosQ^λ^N ^λ^B ^N15^*→ *cosQ^λ^N ^λ^B ^N15^*	N15	11.1 [3.8]	1.88 [0.37]
2	*cosQ^λ^N ^λ^B ^N15^*→ *cosQ^λ^N ^λ^B^λ^*	N15	13.5 [8.3]	1.62 [0.76]
3	*cosQ^λ^N ^λ^B ^λ^*→ *cosQ^λ^N ^λ^B^λ^*	λ	17.7 [4.7]	2.20 [0.44]
4	*cosQ^λ^N ^λ^B^λ^*→ *cosQ^λ^N ^λ^B^N15^*	λ	22.4 [3.9]	2.34 [0.35]

^a^ The host bacteria were MF4944 (line 1), MF4945 (line 2), MF4946 (line 3), and MF4947 (line 4)

^b^ [sem] is the standard error of the mean for 3 or more independent observations.

#### Packaging specificities of λ, 21 and N15

The packaging specificities of λ and 21 are due to the TerS-*cosB* interaction at the start of DNA packaging. To study N15’s packaging initiation specificity, a set of three dilysogens was constructed in which the passive prophage’s upstream initiation *cos* was *cosQ^λ^N^λ^B^λ^* (MF4948), *cosQ^λ^N^λ^B^21^* (MF4949), or *cosQ^λ^N^λ^B^N15^* (MF4950). In each strain the downstream termination *cos* was *Q^λ^N^λ^B^λ^*. These dilysogens were then lysogenized with the λ-, 21-, or N15-specific helper phages. As in previous helper packaging experiments, the λ- and 21-specific terminases failed to package each other’s chromosomes (Table 6). Further, neither the λ-specific or 21-specific helpers packaged the N15-specific chromosome, suggesting that N15 has a unique packaging specificity. The inability of λ and 21 to package N15-specific chromosomes is not unexpected, given the apparent simplicity of *cos^N15^* compared to the elaborate structures of *cos^λ^*and *cos^21^*. The possibility remained that N15 and λ might have the same packaging specificity, but that λ terminase was unable to utilize *cosB^N15^* because of the lack of I1, R2 and R1. In previous work, it was shown that several *Nu1* missense mutations affecting TerS^λ^ (gpNu1) suppressed a variety of *cosB^λ^* defects. The *Nu1ms1* mutation which causes the TerS change L40F, for example, suppresses a number of *cosB* mutations, including the Δ[R2,1] mutation. The Δ[R2,1] deletion takes out *cosB^λ^* bp 92–164, a segment containing R1, R2 and part of I1 [62]. Because *Nu1ms1* terminase sponsors packaging of DNA containing only R3 of *cosB^λ^*, we wondered if *Nu1ms1* terminase would sponsor packaging of N15-specific DNA. We used the N15-specific, λ-specific, and *Nu1ms1* helper phages to ask if *Nu1ms1* terminase could sponsor packaging of N15-specific chromosomes in MF4950. While the N15-specific helper gave a yield of 3.5 prophages/induced lysogen, the prophage yields were 2.9 x 10^-4^ and 1.35 x 10^-4^ for the λ wild type and λ *Nu1ms1* phages, respectively. As observed above with other dilysogens, the N15-specific helper packaged λ-specific chromosomes with reasonable efficiency, i.e., about 50% the efficiency of packaging self-specific N15 chromosomes. In sum, N15 has an asymmetrically unique packaging specificity, with N15 DNA’s specificity being distinct from those of λ and 21, but surprisingly, N15-specific terminase is able to package λ DNA reasonably efficiently.

**Table 6:** Packaging specificities of phages λ, 21 and N15^a^.

Prophage Yields			
	Helper’s Specificity		
Passive Prophage’s Specificity	λ	21	N15
λ	1.75	8.1 x 10^-4^	0.45
	[0.55]^b^	[1.7 x 10^-4^]	[0.19]
21	1 x 10^-4^	1.24	3.5 x 10^-4^4
	[5 x 10^-5^]	[0.11]	[6.5 x 10^-5^]
N15	5 x 10^-4^	2 x 10^-4^	2.5
	[1.5 x 10^-4^]	[6.5 x 10^-5^]	[0.36]

^a^ The dilysogen strains were MF4948 (*cos* ^*λ*^→*cos* ^*λ*^), MF4949 (*cos* ^*21*^→*cos* ^*λ*^) and MF4950 (*cos* ^*N15*^→*cos* ^*λ*^).

^b^ [sem] is the standard error of the mean for 3 or more independent observations.

## Discussion

The goal of this work was to use helper packaging experiments to study N15’s packaging specificity, using λ N15^hy4^ [58]. For helper packaging experiments, the λ-versus-N15 cohesive end difference was a complication. That is, the 12 base-long cohesive ends of λ and N15 differ at bp 9 and 12, and initial helper packaging experiments generated chromosomes with a λ/N15 cohesive end mismatch. We found that chromosomes with mismatched cohesive ends are able to cyclize, and the mismatch persists past replication of the chromosome, in at least some infections. The cohesive end mismatch is correlated with a roughly fourfold reduction in viable phage yield. Burst size studies suggest that TerL^λ^ acts with equal efficiency at *cosN^λ^*and *cosN^N15^*, and that *cos* ^*N15*^ is efficiently utilized for packaging termination. The preliminary helper packaging experiments produced progeny phages, enabling helper packaging experiments on N15’s packaging specificity which did not generate mismatched chromosomes. N15’s DNA packaging specificity was found to be distinct from those of λ and 21. That is, neither λ nor 21 packages N15 chromosomes and N15-specific terminase does not sponsor 21 DNA packaging. Surprisingly, N15-specific terminase packages λ DNA at about 50% efficiency.

### Functional asymmetry of the cohesive ends of *λ*-like phages

*cosN^λ^*, while showing significant two-fold rotational symmetry, is remarkably asymmetric functionally, as follows. An early study of *cosN* mutations found that many mutations affecting bp 7–12 in the right half of *cosN* had minimal phenotypic effects, whereas mutations affecting symmetric bp in the left half of *cosN* have severe phenotypic effects [60]. For example, the G2C mutation reduced virus yield about 10-fold, but the symmetrical C_11_G change had no phenotypic effect. Subsequent biochemical *cos* cleavage experiments confirmed that *cosN* is functionally asymmetric, and further showed that the asymmetry reflected the proximity of *cosN*’s right half to *cosB* [34]. Studies on the importance of the λ *cosN*-to-*cosB* spacing also indicate that the TerS-R3 interaction precisely positions TerL with respect to the *cosN* nicking sites for proper nicking by the TerL endonuclease [33,36]. These results suggest that anchoring of TerL^λ^ to *cosB* makes bottom strand nicking relatively insensitive to base pair changes. The functional asymmetry of *cosN* correlates well with natural *cosN* sequence variation, as follows. The cohesive end sequence of ϕD326, determined long ago [63], differs from *cosN* ^*λ*^ at positions 9 and 12, as does N15. Similarly, *Salmonella* phage Gifsy-1 differs from λ at *cosN* bps 8, 9, and 11. In contrast, the two base pairs flanking the nick positions, 5’-CG-3’ and 5’-CG-3’, are highly conserved, indicating that there is selection against changes of these bps. Similarly, natural variation in *cosQ*, the packaging termination signal, is not apparent. In sum, the results indicate that sequence maintenance for *cosQ* and the left half of the cohesive end sequence is under selection but maintenance of bp positions 7–12 is not.

### Genetic consequences of the λ *versus* N15 cohesive end mismatch

Our experiments show phages carrying λ chromosomes with mismatched cohesive ends, at bp positions 9 and 12, are able to circularize and replicate. For such phages, there is a modest, four-fold decrease in yield. The reduced yield is not due to effects of *cosN* bp changes on terminase efficiency, as both λ and N15^hy4^ terminases sponsor robust virus yields with either *cosN* ^*λ*^ or *cosN* ^*N15*^. We speculate that the reduced yield of viable phages is due to either inefficient cyclization or lethal attempts at mismatch repair. One candidate for repair of the mismatch is the 5’-to-3’ exonuclease of DNA polymerase I, which could carry out nick translation through the mismatched bp. In at least some of the successful infections by phages with mismatched cohesive ends, the mismatch persists through replication of the parental chromosome, since some singly infected cells yield both *cosN^λ^* and *cosN^N15^* progeny phages (Table 2). These results are surprising, since bp position 12 is adjacent to the bottom strand nick which presumably must be ligated prior to replication fork passage. The λ and N15^hy4^ terminases, with TerS^λ^ and chimeric TerS^N15hy4^ subunits, respectively, do not show *cosN* specificity. For both, the large subunit is TerL^λ^ which must be appropriately positioned to nick *cosN*. Given the functional asymmetry of *cosN^λ^*, efficient cleavage of both *cosN^λ^* and *cosN^N15^* is as expected.

### Packaging specificity in the N15-like phages

N15 is an unusual λ-like phage in having a simple *cosB* containing a single critical TerS binding site, R3^N15^. R3^N15^ extends from N15 bp 48–59, and is positioned similarly to R3^λ^. In *cosB* ^*N15*^, there is a proposed additional remote site, “rR2”, in opposite orientation to the critical R3^N15^ sequence and located in the *1* gene at bp 249–260 [58]. The rR2 sequence strongly matches that of R3^N15^ and is found in all N15-like prophages with the simple *cosB* [58]. The rR2 sequence is not critical for N15 growth. Though not essential for plaque formation, rR2 is speculated to contribute to viral fitness for N15 and its close relatives [58]. Another group of prophages, typified by Monarch, has a strong match to R3^N15^ and TerS^N15^’s recognition helix, suggesting a shared packaging specificity (Fig 2). The Monarch group, however, does not have the simple *cosB* ^*N15*^. Rather *cosB* ^*Monarch*^ is analogous to *cosB* ^*λ*^, with three R sequences and an IHF binding site between R3 and R2, and the rR2 sequence is not present.

Comparing the R3 sequences of 21, N15, Monarch, and λ leads to some conclusions about TerS-R3 interactions (Fig 2). The strong identity of R3^N15^ and R3^Monarch^ is apparent, as is the lack of similarity of R3^N15^ and R3^Monarch^ to R3^λ^ and R3^21^. In R3^λ^ and R3^21^, bp 56 and bp 59 are critical for TerS recognition [56]. R3^λ^ bp 58 is also important for TerS^λ^ recognition [55]. R3^N15^ was identified by the packaging effects of two 6 bp-long block mutations, m1 and m2. In m1 and m2, bp 48–53 and 54–59, respectively, were scrambled, as follows. In m1, all six bp were changed–GAGGTT→TGTAGG, and in m2, four were changed-GTTGTT→TGTTGT [58]. The *cosB* ^*N15*^m1 and m2 mutations reduced λ N15^hy4^’s yield by about 7-fold and 100-fold, respectively, indicating bp 54–59 are most important for recognition. That bp 54 to 59 include bp important for TerS interactions agrees with the locations of *cosB* ^*λ*^ and *cosB* ^*21*^ bp 56 and 59, which are important bp for recognition by TerS [56].

Why can N15-specific terminase package λ DNA, but not 21 DNA? Looking at the R3 alignment gives hints, but first it is important to consider the validity of the alignment in Fig 2A. In the alignment, each sequence is equidistant from bp 1, the first base of the left cohesive end. It is possible to shift the N15 and Monarch sequences to increase the sequence identity to R3^λ^ and R3^21^. However in λ, the *cosN*-to-R3 distance is critical, because shifting the distance by inserting or deleting more than one or two bp lowers virus yield and results in improper nick introduction in *cosN* [33,36]. These results indicate that when N15-specific terminase sponsors packaging of λ DNA, TerS^N15hy4^ anchors TerL^λ^ in a manner that closely matches TerS^λ^ anchoring of TerL^λ^. In the *cosB* alignment, R3^N15^ and R3^Monarch^ have a match to the R3^λ^ TA bp 56, an important TerS^λ^contact, R3^λ^ bp 56 also plays a role in λ/21 phage specificity, as follows. Genetic analysis indicates that Glu24 of the TerS^λ^ recognition helix clashes with GC bp 56 of R3^21^, accounting in part for the inability of λ terminase to recognize 21-specific DNA (Fig 2B). The genetics also indicated that Glu24 of TerS^λ^ makes a favorable contact with TA bp 56 of R3^λ^. We speculate that, in the case of N15’s recognition helix, perhaps the Val22 residue, at the position analogous the TerS^λ^’s Glu24, makes a favorable contact with the bp 56 TA bp of *cosB* ^*λ*^, but is unable to make a favorable contact with the GC bp 56 of R3^21^. It is also not clear whether the inability of λ terminase to package N15 DNA is due to a defect in *cosN* cutting, or in a post-cleavage defect such as formation of complex I. More research is required to understand these relationships.

### *cosB* architecture and function

The likely shared packaging specificity of phages N15 and Monarch, and the finding that N15 packages λ DNA raise further interesting questions about *cos* structure and terminase recognition. The interaction of TerS^λ^ with *cosB* ^*λ*^ includes a proposed IHF-assisted DNA hairpin that juxtaposes the major grooves of R3 and R2 for docking by dimeric gpNu1 [20]. The strong similarity of *cosB* ^*λ*^ and *cosB* ^*Monarch*^ suggests a hairpin-containing nucleoprotein structure also forms at *cosB* ^*Monarch*^. It is interesting that the TerS^N15^ DNA binding domain forms a tight dimer [58], even though *cosB* ^*N15*^ is much simpler than *cosB* ^*Monarch*^. Does TerS^N15^ form a similar hairpin structure at *cosB* ^*N15*^, even though there is neither an IHF site nor an R2-equivalent? Elsewhere we propose that *cosB* ^*N15*^ is derived from *cosB* ^*Monarch*^ by unequal crossing over between R3 and R1. If so, the TerS^N15^ would be derived from TerS^Monarch^, and might retain the ability to interact with *cosB* ^*Monarch*^ in a manner similar to TerS^λ^ docking at *cosB* ^*λ*^. A related issue is packaging of λ DNA by N15-specific terminase. Does TerS^N15hy4^ utilize all three R sequences of *cosB* ^*λ*^ when packaging λ DNA? These interesting questions await further studies.

What evolutionary steps account for the packaging systems of the λ-like phages? It seems clear that λ and 21 are descended from a common ancestor phage, as the basic *cos* structure is preserved. We earlier presented one possible path, in an early ancestor virus where a single R sequence and a terminase with low sequence specificity acquired greater packaging specificity by acquisition of three R sequences [64]. A scenario for N15 is that N15 has undergone a simplification process, by which a Monarch-like ancestor phage with three R sequences has lost two, and the IHF site, by unequal crossing over between R3 and R1. One would think that such an event would result in a major fitness reduction. It is possible however that the simplification and subsequent refinement, such as the acquisition of rR2, might take place in the prophage state, where fitness for lytic growth is moot [58,65].

### Genetic consequences of the asymmetric, shared packaging specificities of λ and N15

The chromosomes of many bacteriophages, including the λ-like phages, are mosaics of DNA segments, indicating that extensive horizontal exchange is part of a phage’s evolutionary history [66]. Enteric bacteria frequently harbor λ-like prophages, so that an infecting λ-like phage has a strong likelihood of replicating in the presence of another λ-like phage chromosome. Of course several significant consequences may result from such an interaction. If a prophage shares the packaging specificity of the infecting phage that enters the lytic cycle, then the packaging machinery of the infecting phage will initiate packaging at the prophage *cos*. For the λ-like prophages, *cos* is located roughly in the center of the prophage, so that the packaging initiation encapsidates a DNA segment that will include adjacent bacterial DNA, stalling the motor when the head shell is full, which in turn produces a tailless, non-infectious particle. Production of such inactive phage structures reduces the production efficiency of viable progeny phages. One might think therefore, that it would be advantageous for λ-like phages to have diverse packaging specificities. So far, only modest diversity is observed, though not many examples have been studied. Among characterized λ-like phages with related *cos* structures, the three packaging specificities of λ, 21 and N15 have been documented. Other natural isolates, such as phages 434 and ϕ80, share λ‘s packaging specificity. N15 is an interesting case, as N15’s packaging machinery retains the ability to package λ DNA, even though N15 has a significantly divergent DNA recognition system.

A second interaction between λ-like phages is recombination. If a packaging event initiated at a prophage *cos*, is accompanied by a downstream crossover between prophage and lytic phage DNAs, the recombinant is a novel progeny phage that contributes to genetic diversity. Some such genetic exchanges generate recombinants that are more robust in a changing environment. In the case of a cohesive end mismatch, such as in the present λ versus N15 situation, recombination may produce less fit progeny phages. Because the cohesive end mismatch does not persist, the reduced viability of a recombinant phage is transient.

### DNA recognition by TerS

TerS molecules interact with their recognition sites through their N-terminal DNA binding domains, and with TerL through their C-terminal domains. These two specificity domains are separated by the long α-helical core domain. In principal, phage diversity might be generated by swapping of the TerS DNA recognition domain for that of any other DNA binding protein with an asymmetric recognition site. In the present case, we note that both the *cos* and TerS of N15 are recognizably evolutionarily related to those of phage λ [58]. Phage 21’s packaging specificity determinants also share a common ancestor with λ [56]. N15 and 21 were chosen for study as interesting variants of the λ paradigm, and as such these phages do not shed light on extreme possibilities for specificity swapping. We note that in a more systematic study in P22-like phages, considerable horizontal transfer of TerS gene segments has occurred, not only between highly diverged members of the P22 family, but also has occurred between P22-like phages and phages outside the P22-like group [16]. Acquisition of a TerS segment from a non-phage source such as a transcriptional regulatory system has not been observed. Surely further sequence acquisition and study will add to what we know about this issue.

## Materials and Methods

### Media

Luria broth (LB), LB agar, tryptone broth (TB), tryptone broth agar (TA), and tryptone broth soft agar (TBSA) were prepared as described [67], except TB, TA, and TBSA were supplemented with 0.01 M MgSO_4_. For phage infections, TB was supplemented with 0.2% maltose. When required, ampicillin, chloramphenicol, and kanamycin were added at 100 μg/mL, 10 μg/ml, and 50 μg/mL, respectively.

### Bacteria, phages and plasmids

These are listed in Table 1. A list of dilysogens constructed for this work is in S1 Table.

### Microbiological methods

Phage crosses and infections were done using standard protocols [67].

### Helper phages

The helper phages used were in the thermo-inducible λ-P1:5R *cI*857 Kn^R^ *nin*5 genetic background. In λ-P1:5R *cI*857 Kn^R^ *nin*5, the DNA segment containing the λ site-specific recombination system is replaced by the phage P1 plasmid replication and partitioning segment, so the helper phage forms a plasmid prophage [68]. The Kn^R^ marker in λ-P1:5R *cI*857 Kn^R^ *nin*5 is a substitution for λ DNA between the two λ SalI sites [69]. The SalI sites are located in (1) the *bet* gene and (2) distal to *gam*, so that the helper phages are *red* ^*-*^ *gam* ^*-*^. When growing in a *recA* ^-^ host, *red* ^*-*^ *gam* ^*-*^phages are unable to generate concatemers through rolling circle replication or recombination [29]. Consequently, the helper phage yield is low, about 10–20 phages/cell in the experiments reported here. For simplicity, λ-P1:5R *cI*857 Kn^R^ *nin*5 will simply be designated λ; this phage was used as the helper with λ packaging specificity. λ 21^hy51^ [59] and λ N15^hy4^ [58] were the 21- and N15-specific helpers, respectively.

### Dilysogens

Dilysogens were constructed with two *recA* ^-^ strains with deleted prophages, MF713 and MF532 (Table 2). The λ *b511* prophage of MF713 is deleted for a DNA segment extending from the bacterial *gal* operon thru the prophage early genes, including *cI*, while the segment containing *cos*, the head and tail genes, the *b511* marker and *attΔ●B* are present. The genetic structure of MF713 is:

$$\Delta [gal\text{---}\underline{attL-cl-S]-cos^{\lambda }-\text{//}-b511\;att\Delta •B}\text{--}bio\text{-}$$

where λ genes are underlined, slashes indicate the long DNA stretch of the λ head and tail genes, dashed lines indicate *E*. *coli*. Lysogenization of MF713 by λ *imm^434^ ind* ^*-*^ *cos_i_* generates the following genetic structure:

$$\Delta [gal\text{---}\underline{attL\text{-}cl-S]\text{-}\underline{cos^{\lambda }\text{-//-}attP\text{-}imm^{434}-cos_{i}}-\text{//}-attR}\text{--}bio\text{-},$$

where *cos_i_* indicates that the downstream *cos*, at which termination occurs, may have λ, 21 or N15 sequences. The passive packageable prophage is double-underlined. Care was taken to exclude isolates with two copies of the added prophage. For MF713 (λ *imm* ^*434*^ *cos_i_*), we confirmed that the chromosomes produced by helper packaging of the passive prophage carried the *b511* marker. Generally, 10 isolates were tested by a PCR assay or a test for a *b511* phage’s inability to lysogenize.

The λ *imm^434^* prophage of MF532 is deleted rightwards from *Nu1* through the head and tail genes, *attR*, and bacterial DNA including *bio*, *uvrB* and *chlA*. The genetic structure of MF532 is:

$$gal\text{--}\underline{attL-imm^{434}\text{-}cos^{\lambda }-\Delta [Nu1-\text{//}-attR}-bio\text{--}uvrB-chlA]$$

Lysogenization of MF532 by λ *imm^21^ cos_i_* gives the genetic structure:

$$gal\text{--}\underline{attL\text{-}imm^{21}\text{-}cos\underline{{}_{i}-\text{//}-attP\text{-}imm^{434}\text{-}c}os^{\lambda }\text{-}\Delta [Nu1-\text{//}-attR}-bio\text{--}uvrB-chlA]$$

where the passive packageable prophage is double-underlined. The *cos* at which packaging is initiated, *cos*_i_, was either *cos^λ^*, *cos^21^* or *cos^N15^*. The *cos* ^*21*^ is from 21^hy33^, which also has gene *1* and a chimeric *2/A* gene. The *N15^hy4^* provided *cos^N15^*. Care was taken to ensure that dilysogen candidates with more than two prophages were eliminated.

For MF532 dilyogens, the upstream prophage carried the immunity of phage 21 (*imm* ^*21*^) and *cos_i_*. The downstream prophage of MF532 carries *imm^434^* and *cos* ^λ^. Helper packaging of the passive prophage of an MF532 dilysogen generates virions carrying chromosomes that are *imm^434^*. The immunity of 10 isolates was tested to confirm this expectation.

#### MF611 (Tables 3 and 6)

To generate this dilysogen set, the *recA1 E*. *coli* strain MF611 was sequentially lysogenized by the two phages under study. Then helper phages were added. The genetic structure of dilysogens was determined by examining the genetic content of helper-packaged passive prophages. In all cases care was taken to show that isolates with more than two prophages were excluded from experiments.

#### MF3510 (Table 5)

MF3510 is a *galK* ^*-*^derivative of MF611. Phages used carried *gal* or *cat* markers, and examination of packaged passive prophages was used to eliminate isolates with more than two prophages. In most cases, for all three dilysogen sets, multiple independent dilysogen isolates were used in experiments.

#### Helper phages

The helper phage genetic background was λ-P1:5R Kn^R^ *cI857 nin5*, and this phage was the λ- specific helper phage. The 21-specific helper phage was a recombinant containing the left chromosome end 21^hy51^, which includes *cosB^21^* and a chimeric small subunit gene in which the 5’-half was from the 21 *1* gene and the 3’ half was from *Nu1*. The N15-specific helper, λ-P1 N15^hy4^, is described in Results. For simplicity, these three helper phages will simply be designated as the λ-, 21-, and N15-specific helpers, resp.

#### Helper packaging protocol

Strains were grown overnight in LB+kanamycin, in standing culture, at 31C, and 0.2 ml was used to inoculate 5 ml of LB. Cultures were shaken at 31C for 2 hrs. An aliquot was removed and plated to determine the cell titer. Helper phages were induced by shifting to 42C for 15 min, and the cultures were shaken at 37C for an additional 70 min. Lysis was completed by addition of CHCl_3_, debris removed by centrifugation, and the lysates titered at 37C. Helper phages form clear plaques and phages bearing passive prophages form turbid plaques. For lysates with low levels of passive prophage-containing virions, dilutions were plated on C600(λ). If the progeny of phages with passive prophages were to be subjected to further analysis, the indicator strain was MF1427, an *E*. *coli* C strain. The level of defective λ-like prophage material in *E*. *coli* C is very low, and no *cosN^λ^* is present, so that concern about recombination with defective prophage DNA was eliminated.

## Supporting Information

S1 Table Dilysogens used in Helper Packaging Experiments. (DOCX)
