# Supplementary material for: DNA Packaging Specificity of Bacteriophage N15 with an Excursion into the Genetics of a Cohesive End Mismatch
Source: PLoS One. 2015 Dec 3;10(12):e0141934. doi: 10.1371/journal.pone.0141934 (PMC4669245; doi:10.1371/journal.pone.0141934)
Supplement: S1 Table — (DOCX) [file pone.0141934.s001.docx]

| S1 Table Dilysogens for Helper Packaging Experiments | | | | |
| --- | --- | --- | --- | --- |
|  |  |  |  |  |
| Strain | Experiment: | Bacterium | Prophage 1 | Prophage 2 |
| MF4942 | Table 3 | MF611 | φ72: *cosQλNλBλ* | φ1201: *cosQλNN15Bλ* |
| MF4943 | Table 3 | MF611 | φ72: *cosQλNλBλ* | φ1203: *cosQλNλBλ* |
| MF4944 | Table 5 | MF3510 | φ1227: *cosQλNλBN15* | φ1197: *cos QλNλBN15* |
| MF4945 | Table 5 | MF3510 | φ1227: *cosQλNλBN15* | φ1220: *cosQλNλBλ* |
| MF4946 | Table 5 | MF3510 | φ1225: *cosQλNλBλ* | φ1220: *cosQλNλBλ* |
| MF4947 | Table 5 | MF3510 | φ1225: *cosQλNλBλ* | φ1197: *cos QλNλBN15* |
| MF4948 | Table 6 | MF611 | φ1225: *cosQλNλBλ* | φ1220: *cosQλNλBλ* |
| MF4949 | Table 6 | MF611 | φ1221: *cosQλNλB21* | φ1220: *cosQλNλBλ* |
| MF4950 | Table 6 | MF611 | φ1227: *cosQλNλBN15* | φ1220: *cosQλNλBλ* |
